# Molecular docking analysis of potential compounds from an Indian medicinal soup "kabasura kudineer" extract with IL-6

**DOI:** 10.6026/97320630017568

**Published:** 2021-05-31

**Authors:** S Saleem Basha, Dhirendra Tripathi, Sravanthi Koora, K Satyanarayana, Selvaraj Jayaraman

**Affiliations:** 1 Dept of Medical Biochemistry, School of Medicine, Haramaya University, Harar Campus, Ethiopia; 2 Department of Otorhinolaryngology, Government Medical College, Shivpuri, Shivpuri- 473638; 3 Department of Pharmacology, Government Medical College Siddipet-502103, Siddipet, Telangana; 4 Department of Biochemistry, Government Medical College Siddipet, Siddipet 502103 Telangana India; 5 Department of Biochemistry, Saveetha Dental College and Hospitals, Saveetha Institute of Medical and Technical Sciences, Chennai-600 077, India

**Keywords:** Molecular docking, IL-6, CoVid-19, "kabasura kudineer"

## Abstract

The use of "kabasura kudineer" (liquid soup made from Indian medicinal plants) for combating COVID-19 has been common in the states of Tamilnadu and Puducherry, India during the pandemic. Therefore, it is of interest to document the molecular docking analysis of IL-6 inhibitors with potential antiviral compounds from "kabasura kudineer" extract. We show the optimal binding features of gallic acid and luteolin with the Interleukin-6 protein for further consideration.

## Background

Symptoms of the novel corona virus are similar to normal flu linked to CoVid-19 in structure and cause [1-8]. Jing Liu et al. [7] reported that CoVid-19 patients sustained decrease in the proportion of lymphocytes with increase in the inflammatory cytokines (interleukin) in the peripheral blood. It is also known that IL-6 binds with gp130 to initiate downstream signal transduction, gene expression, and intracellular signal transduction [9-20]. The design, development and evaluation of compounds to combat the viral pandemic are gaining momentum. The use of "kabasura kudineer" (liquid soup from Indian medicinal plants) for combating CoVid-19 has been common in the states of Tamilnadu and Puducherry during the pandemic. Therefore, it is of interest to document the molecular docking analysis of compounds from an Indian medicinal soup "kabasura kudineer" extract with IL-6.

## Material and Methods:

Molecular docking analysis was performed using the Maestro 11.4, Schrodinger 2017-4 [21][22 - check with author].

## Ligand preparation:

38 reported antiviral compounds [23-26] were used as ligands for the molecular docking analysis. PubChem and Drug Bank were used to download the 2D structures for the ligands. LigPrep module (Schrodinger, LLC, NY, USA, 2009) was utilized from the Maestro developer to design and create the 3D ligand structures by eliminating salt, adding hydrogen molecules, and ionizing at pH (7.0 +/- 2.0). Energy minimization was performed utilizing OPLS3 force field by utilizing the standard energy capacity of atomic mechanics. A RMSD cut-off at 0.01 Å was used to create the low-energy ligand isomer.

## Preparation of protein structures:

Protein structure of IL-6 (PDB IDs: 3L5I, having resolution < 1.90 Å, R-value free <0.222, R-Value Work <0.181) was downloaded from the Protein Data Bank (http://www.rscb.org) [27]. Assigned bonds orders and hydrogen atoms were added. Water molecules were removed within 3Å of HET groups [20]. OPLS3 force field in Schrodinger, LLC, NY, USA, 2009 was used for energy minimization [28]. The receptor grid boxes were generated using Glide's Receptor Grid Generation module at the active site (with the radius of 20 around the crystal structure) of co-crystallized ligand with the computing cubic box of 14.74 x 53.85 x 73.53 .

## Molecular docking

Flexible docking with GLIDE Extra precision (XP) convention was used for calculating the binding affinity and ligand efficiency as an inhibitor of Corona virus target [29-30]. Maestro interface (Schrodinger Suite, LLC, NY) was used as the visualization tool for docked ligands.

## Results and Discussion:

**Figure 1:**
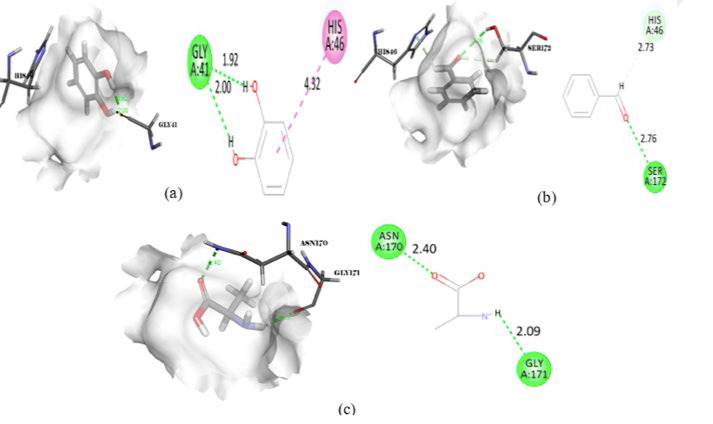
Docking interactions of gallic acid (CID_370) with 3L5I.

**Figure 2:**
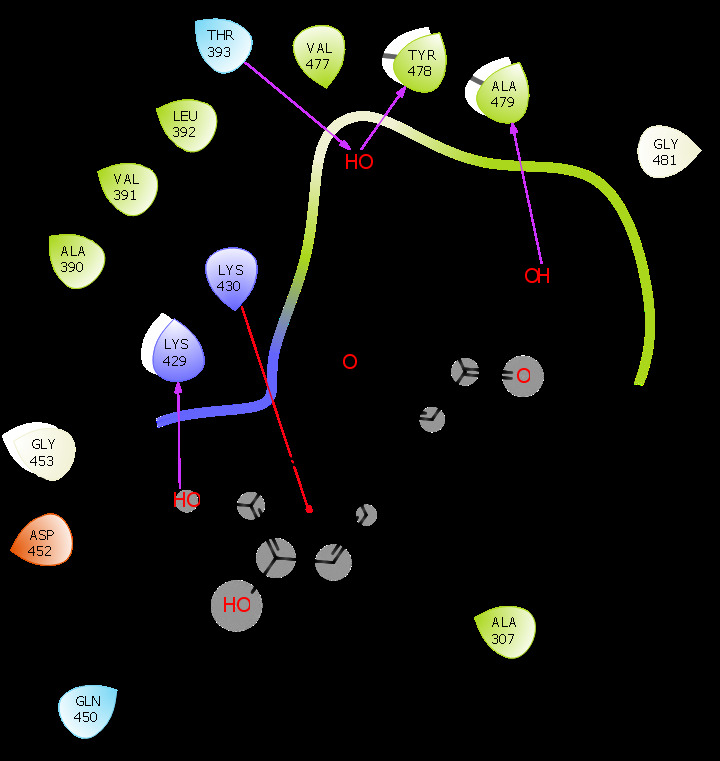
Docking interactions of luteolin (CID_5280445) with 3L5I.

The use of "kabasura kudineer" (liquid soup from Indian medicinal plants) for combating COVID-19 has been common in the states of Tamilnadu and Puducherry during the pandemic. Therefore, it is of interest to document the molecular docking analysis of IL-6 inhibitors with potential antiviral compounds from "kabasura kudineer" extract (Table 1 - see PDF). The potential binding residues in IL-6 are TYR, ALA, VAL, LYS, TRY, THR, and ASP. 38 molecules were docked with IL-6 and ranked based on their dock score. Compounds with optimal binding features are given in Table 2(see PDF). Table 2 (see PDF) shows that compounds (gallic acid and luteolin) showed good binding interactions with IL-6 structures compared to known standard compounds. Compound 1 (gallic acid) has one salt bridge interaction with LYS430 and three hydrogen bonding interactions with VAL477, TRY478, and ALA79 (Figure 1). It also has more lipophilic interactions and non-bonded interactions against IL-6. When comparing to other compounds including the standard anti-HIV drugs, gallic acid has a more binding affinity towards IL-6 and it showed best hydrogen bonding interactions. Compound 2 (luteolin) has one pi-stacking interaction with LYS430 and four hydrogen-bonding interactions with LYS429, THR393, TYR478, and ALA479 (Figure 2). It also has more lipophilic interactions and non-bonded interactions against IL-6. When comparing to other compounds including standard anti-HIV drugs, Luteolin has good binding affinity, hydrogen bond towards IL-6. Thus, data show that they are involved in non-bonded interaction with IL-6.

## Conclusion:

We report that gallic acid and luteolin have good binding features with the Interleukin-6 protein for further consideration. Thus, kabasura kuduneer extract containing gallic acid and luteolin used in combating COVID-19 is of importance.
